# Thermal insulation performance and environmental assessment of vermiculite and agricultural residue based composites for the Kahramanmaras climate

**DOI:** 10.1038/s41598-026-40255-6

**Published:** 2026-02-28

**Authors:** Mustafa Eken, Ayşenur Gürgen, Aytaç Dinçer

**Affiliations:** 1 Elbistan Vocational School of Higher Education, Department of Construction Technology, Kahramanmaraş Istiklal University, Kahramanmaras, Turkey; 2 Department of Industrial Engineering, Engineering and Natural Sciences Faculty, Osmaniye Korkut Ata University, Osmaniye, 80000 Turkey; 3 Department of Civil Engineering, Faculty of Engineering and Architecture, Sutcu İmam University, Kahramanmaras, Turkey

**Keywords:** Thermal insulation, Sustainable composites, Agricultural waste, Vermiculite-epoxy, Energy efficiency, Fire resistance, ANOVA, Energy science and technology, Engineering, Environmental sciences, Materials science

## Abstract

Thermal insulation plays a crucial role in improving energy efficiency in buildings, particularly in regions with high residential and industrial energy demand. However, conventional petrochemical-based insulation materials raise significant environmental concerns, highlighting the need for sustainable and renewable alternatives. In this study, bio-based composite insulation materials were developed using locally available agricultural residues—stubble, sunflower stalks, corn cobs, corn stalks, and olive pits—incorporated into a vermiculite–epoxy resin matrix. The developed composites exhibited low thermal conductivity values of approximately 0.041 W/mK, comparable to that of expanded polystyrene, while demonstrating enhanced fire resistance due to the presence of vermiculite. Life cycle assessment and experimentally based CO₂ savings analyses revealed substantial reductions in both embodied and operational carbon emissions. The utilization of agricultural waste not only prevents open-field burning but also reduces environmental impacts and supports local economies. Statistical analysis using ANOVA confirmed that the type of agricultural residue significantly influences the thermal and mechanical performance of the composites. Overall, the results demonstrate that the proposed bio-based composites provide a balanced combination of thermal, mechanical, and fire-resistant properties, offering a sustainable, cost-effective, and regionally adaptable solution for energy-efficient building insulation, particularly in climatic conditions similar to those of Kahramanmaras, Turkey.

## Introduction

The rapid growth of the global population, together with the accelerating pace of industrialization and urbanization, has led to a significant increase in global energy demand^1,2^. This trend is particularly pronounced in developing countries, where unplanned urbanization and insufficient consideration of energy efficiency criteria result in further increases in energy consumption^3^. In Turkey, the construction sector accounts for approximately 40% of total energy consumption, with a substantial share attributed to the thermal performance of buildings^4–9^. Therefore, the implementation of effective thermal insulation solutions is critical not only for reducing energy consumption but also for mitigating carbon emissions and limiting environmental impacts.

In recent years, environmental sustainability has become a key criterion in the selection of building materials. Conventional insulation materials are predominantly derived from petrochemical sources, and their production processes are energy-intensive and associated with adverse environmental effects^10–14^. This situation has necessitated the development of environmentally friendly insulation solutions based on renewable resources. Agricultural wastes such as corn cobs, wheat stalks (stubble), sunflower stalks, rice straw, and various husks have emerged as promising alternatives due to their abundance, renewability, and inherent thermal insulation properties^15–25^. Similarly, natural fibers including jute, flax, and hemp have been extensively investigated for high-performance thermal insulation applications^21–23^.

The selected agricultural residues—stubble, sunflower stalks, corn cobs, corn stalks, and olive pits—were strategically chosen based on their local availability, documented thermal insulation and mechanical performance, and their contribution to sustainable waste valorization. Unlike previous studies that focused on single or limited residue types^1–3^, the present work integrates multiple locally sourced wastes into a vermiculite–epoxy matrix, achieving a multifunctional composite with low thermal conductivity, adequate mechanical integrity, and enhanced fire resistance, while simultaneously promoting regional circularity and reducing CO₂ emissions.

The utilization of these waste-derived or naturally sourced materials provides thermal insulation performance comparable to that of conventional products, while simultaneously reducing CO₂ emissions resulting from open-field burning, thereby preventing environmental pollution and supporting the principles of the circular economy. This environmental contribution is further substantiated through a quantitative life-cycle CO₂ saving analysis (Sect.  3.7), in which the developed composites are benchmarked against conventional insulation materials. The observed life-cycle CO₂ reductions are comparable to or, in some formulations, exceed those reported in recent waste-based insulation studies^1–3^, highlighting the potential of the developed composites to achieve both competitive thermal performance and enhanced environmental benefits.

However, a review of the literature indicates that most studies focus on a single type of agricultural waste or evaluate only limited performance parameters, such as thermal conductivity^15,16,21–23^. Comprehensive studies that simultaneously assess multiple criteria—including thermal insulation, mechanical strength, and fire resistance—remain limited. Furthermore, the systematic evaluation of regional agricultural waste potential has not been sufficiently addressed in existing research^26–28^.

Recent high-impact studies have further demonstrated the growing interest in the valorization of recycled and bio-based wastes for building insulation applications. For instance, composites produced from cardboard waste and date palm fibers with polystyrene additions were reported to achieve thermal conductivity values between 0.085 and 0.104 W/m·K, together with acceptable compressive strength levels, indicating performances comparable to conventional insulation materials^29,30^. However, these investigations also revealed very high capillary water absorption rates, in some cases exceeding 200%, which restrict their applicability primarily to protected or interior environments^29,30^.

Similarly, plant-fiber-reinforced epoxy composites have been shown to provide promising insulation efficiency, with thermal conductivity values as low as 0.069 W/m·K and favorable economic returns based on optimized thickness analyses^31^. Despite these advantages, such systems generally rely on single or limited fiber types and often do not consider the availability of regional agricultural residues or the environmental implications associated with local waste management practices.

Compared with these studies, the present research advances the field by simultaneously utilizing multiple agricultural residues obtained from a specific regional inventory and integrating them within a vermiculite–epoxy hybrid matrix. This strategy not only targets low thermal conductivity, but also seeks to improve fire resistance, mechanical integrity, and environmental compatibility within a single material design. By moving beyond mono-fiber approaches and laboratory-scale demonstrations, the study contributes to bridging the gap between material development and regionally scalable, real-world insulation solutions, thereby enabling broader implementation in sustainable construction practices.

To the best of the authors’ knowledge, no previous study has comprehensively investigated the combined use of multiple region-specific agricultural wastes within a vermiculite-supported epoxy system while simultaneously addressing thermal, mechanical, hygroscopic, and fire-performance criteria.This study therefore positions itself beyond existing waste-based insulation approaches by proposing a multifunctional, regionally grounded composite concept that combines material circularity with practical performance requirements and enables realistic large-scale applicability.

In addition, recent studies emphasise that the incorporation of vermiculite provides significant advantages in enhancing thermal insulation performance and improving the mechanical properties of composite materials^32,33^. In this study, a novel composite thermal insulation material was developed using a vermiculite–epoxy resin matrix incorporating various agricultural wastes, including stubble, sunflower stalk, corn cob, corn stalk, and olive pit, sourced from the Kahramanmaras–Elbistan Plain, one of Turkey’s important agricultural production regions^26–28^. This approach aims to produce a lightweight, fire-resistant, and thermally efficient material while ensuring the effective utilization of local waste resources. The use of stubble contributes to the protection of soil microorganisms that would otherwise be adversely affected by open-field burning and also supports the reduction of greenhouse gas emissions. Moreover, the exclusive use of locally sourced materials enhances the regional adaptability, sustainability, and scalability of the developed composite.

From a sustainable development perspective, the design of building materials that considers environmental performance, resource efficiency, and energy-saving criteria is becoming increasingly important. Accordingly, the composites developed in this study are evaluated not only in terms of thermal conductivity but also with respect to mechanical strength, water absorption, and fire resistance. This comprehensive assessment enables a holistic evaluation of agricultural waste-based composites and provides a comparative analysis of different waste types within a single experimental framework^17,27,28,34,35^. The performance improvements achieved in these parameters are not only material-level advancements but also translate directly into environmental benefits. In particular, lower thermal conductivity and optimized density profiles reduce operational energy requirements during the service life, thereby enabling measurable reductions in life-cycle CO₂ emissions. Simultaneously, the systematic utilization of agricultural residues reinforces sustainable waste management practices and supports regional circularity frameworks.

Recent experimental findings obtained within this study indicate that the proposed composites achieve thermal conductivity levels consistent with insulation-class materials and comparable to those reported in recent recycled and bio-based systems, including the composites described in References^1–3^. However, unlike these previously reported materials, which primarily emphasize thermal efficiency, the present formulation adopts a broader multifunctional strategy through the incorporation of vermiculite and the simultaneous use of multiple region-specific agricultural residues. This approach aims not only to provide competitive insulation performance but also to enhance fire resistance, mechanical reliability, and environmental compatibility within a unified material design. These tendencies highlight the potential of the developed system to move beyond laboratory-scale concepts toward practically applicable and regionally adaptable insulation solutions.

In conclusion, this research presents an original and comprehensive approach to the development of next-generation bio-based composite insulation materials that are regionally available, environmentally sustainable, and capable of meeting multiple performance criteria. The findings offer a significant contribution to the existing literature on sustainable building technologies and provide a foundation for the development of region-specific insulation solutions.

## Materials and methods

### Materials

#### Sunflower stalk

Sunflower is an important industrial crop widely cultivated worldwide and in Turkey, primarily for oil production. In Turkey, sunflower is grown on approximately 500,000–600,000 hectares under both irrigated and rainfed agricultural conditions. The sunflower stalks used in this study were collected from agricultural fields in the Elbistan region. To enable their use in fiber form, the stalks were cut into lengths of 3–5 cm, dried, and subsequently utilized as reinforcement materials in composite production.

In addition, the bulk density and water absorption capacity of the materials were experimentally determined using specimens measuring 50 × 50 mm, in accordance with TS EN 16,023^36^ and TS EN ISO 16,535^37^ standards (see Sect.  2.3). These measurements provided essential information for understanding material behaviour in terms of homogeneity and workability during composite fabrication.

#### Stubble

Stubble refers to the remaining stalks and root residues left in the field after cereal harvesting. In Turkey, approximately 10 million tons of stubble are generated annually from the production of crops such as wheat, barley, and rye. The stubble used in this study was collected from agricultural lands in the Elbistan region, cleaned to remove foreign materials, cut into lengths of 3–5 cm, and dried prior to composite production. These characteristics are critical for predicting the material’s behaviour and performance within the composite structure.

#### Corn cob

Corn cobs are agricultural by-products generated during maize processing and are often left underutilized. Due to their porous structure and low density, corn cobs offer advantages in terms of thermal insulation performance. In this study, corn cobs obtained from local sources were cleaned, cut into pieces approximately 3–5 cm in size, dried, and used as filler materials in the composite system. Their density and water absorption properties were measured to evaluate their contribution to composite design.

#### Corn stalk

Corn stalks are fibrous biomass wastes produced during maize harvesting. Their low density and fibrous structure make them promising candidates for lightweight composite materials with enhanced thermal insulation performance. The collected stalks were cut into lengths of 3–5 cm, dried, and incorporated into the composite mixtures. Their bulk density and water absorption characteristics were experimentally determined to assess their physical performance within the composite matrix.

#### Olive pit

Olive pits are lignocellulosic by-products generated during olive oil production and exhibit a hard and compact structure, making them suitable for composite material applications. The olive pits used in this study were obtained from processing facilities, cleaned to remove residual pulp, ground to sizes of approximately 3–5 cm, dried, and incorporated into the composite mixtures. Their density and water absorption properties were measured and used as reference parameters in composite material design.

### Composite fabrication method and rationale of experimental design

In the composite insulation materials developed in this study, vermiculite was selected as the primary inorganic component due to its low thermal conductivity and high fire resistance. Epoxy resin was used as the binder phase to ensure effective bonding of agricultural waste materials to the matrix, enhance the mechanical integrity of the specimens, and achieve homogeneous dispersion during fabrication.

Sunflower stalks, stubble, and corn stalks were prepared with lengths of 130–140 mm, while corn cobs (25–30 mm in diameter) and olive pits were processed into appropriate sizes. The composite mixtures were produced by combining agricultural waste materials, vermiculite, and epoxy resin at predetermined ratios, as presented in Table 1.

**Table 1:** Component quantities used in composite samples (g).

Sample code	Vermiculite (g)	Sunflower stalk (g)	Stubble (g)	Corn cob (g)	Corn stalk (g)	Olive pits (g)	Epoxy resin (g)
R	150	0	0	0	0	0	150
SS1		25					
SS2		50					
S1		0	25				
S2			50				
CC1			0	25			
CC2				50			
CS1				0	25		
CS2					50		
O1					0	25	
O2						50	

The material proportions were determined based on low to moderate reinforcement levels (approximately 2.5–10 wt%) commonly reported in the literature for bio-based composites^38^. In addition, preliminary trials indicated that high waste contents adversely affected specimen integrity, whereas very low reinforcement levels limited thermal insulation performance. Accordingly, the agricultural waste contents were selected as 25 g and 50 g. These values enabled a comparative evaluation of physical properties such as bulk density and water absorption.

The composite specimens were fabricated using metal moulds with dimensions of 160 × 160 × 40 mm. The production procedure was carried out as follows:

- An epoxy resin layer was applied to the base of the mould.
- The first layer of the composite mixture was placed into the mould.
- Pre-treated plant-based fibers were introduced as an intermediate layer.
- Epoxy resin was applied to each layer to enhance interfacial bonding.
- The specimens were compacted under a pressure of 5 bar.
- The samples were cured in the mould for 24 h and subsequently demolded.

**Fig. 1:**
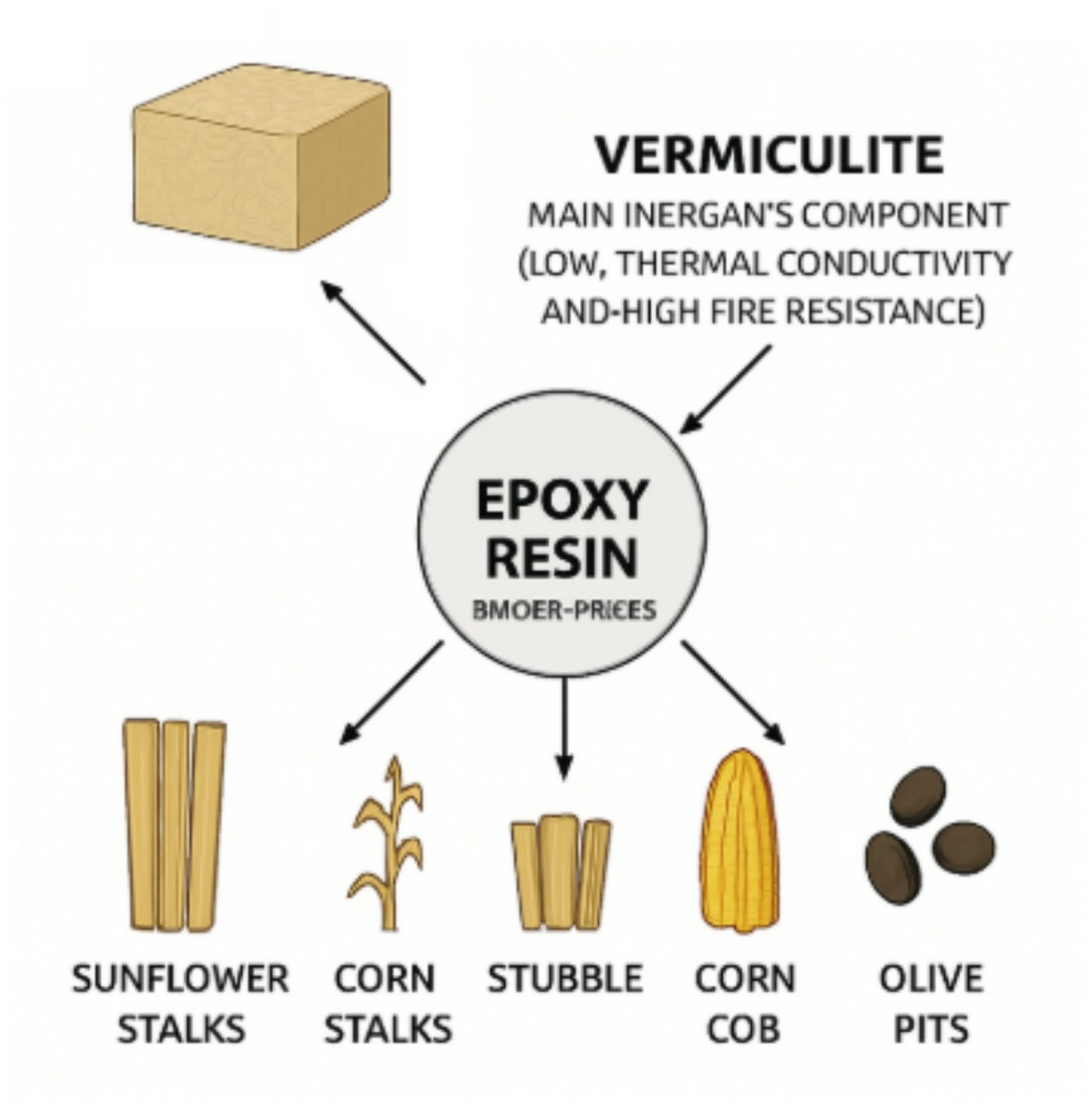
Grouped input materials and processing steps for composite sample production.

This fabrication method was selected to ensure homogeneous material distribution, adequate interfacial adhesion, and overall structural integrity. A schematic illustration of the production process is presented in Fig. 1.

### Bulk density and water absorption

Bulk density and water absorption tests were conducted on specimens with dimensions of 50 × 50 × 30 mm. The specimens were first weighed, then immersed in water for 24 h, and subsequently weighed again. The tests were performed in accordance with TS EN 16,023 ^36^ and TS EN ISO 16,535 ^37^ standards.

### Ultrasonic pulse velocity (UPV)

UPV measurements were carried out on specimens with dimensions of 50 × 50 × 30 mm in accordance with ASTM E494^39^. The wave velocity was calculated using Eq. (1):

1 $$\:V=\frac{S}{t}\times\:{10}^{6}$$

Where; *V* is the ultrasonic wave velocity, *S* is the specimen length (mm), and *t* is the wave transit time (µs).

Three measurements were taken for each specimen, and the average values were used for evaluation.

### Thermal conductivity

The thermal conductivity coefficient (λ) was measured using a KEM QTM-500 device in accordance with ASTM C1113-90 ^40^. The tests were performed on specimens with dimensions of 70 × 130 × 30 mm. Three measurements were taken from each surface, and the average values were reported.

### Mechanical properties

#### Compressive strength

Compressive strength tests were performed on specimens with dimensions of 160 × 160 × 40 mm using a Zwick Roell Z010 universal testing machine at a loading rate of 5 mm/min.

#### Flexural strength

Flexural strength was determined by a three-point bending test with a span length of 150 mm, using an Emic DL 30,000 testing machine, in accordance with TS EN 826 ^41^.

### Fire resistance test

**Fig. 2:**
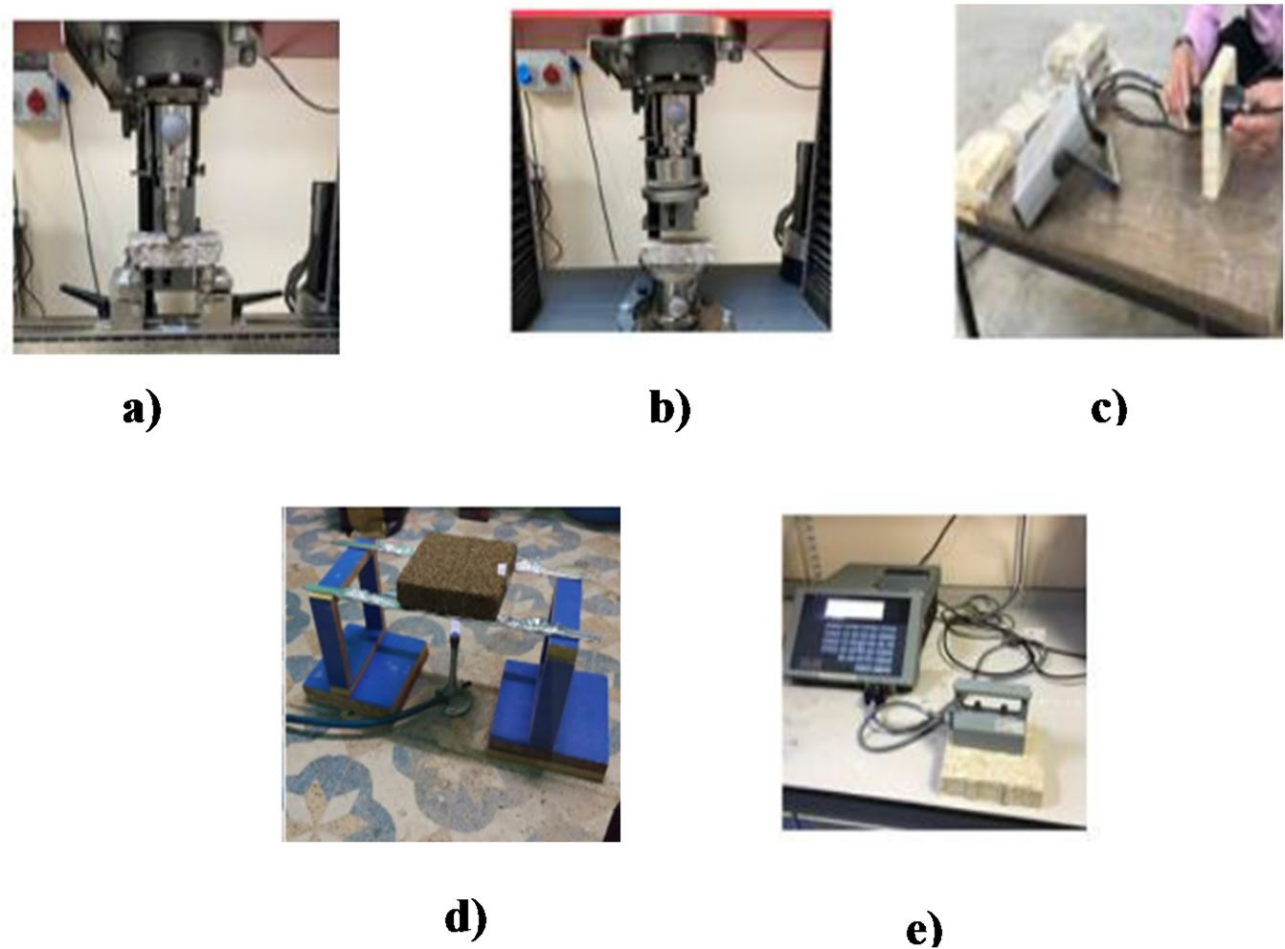
Experimental procedures: (**a**) compressive strength, (**b**) flexural strength, (**c**) ultrasonic pulse velocity (UPV), (**d**) fire resistance and (**e**) thermal conductivity.

Fire behavior was evaluated using a methane burner producing a blue flame with a length of 20 mm. The distance between the flame tip and the specimen surface was set to 10 mm (Fig. 2). The tests were conducted in accordance with TS EN ISO 11925-2:2020 ^42^.

The experimental tests carried out on the fabricated samples are shown in Fig. 2.

### CO₂ and energy savings calculations for bio-based composites

The CO₂ savings achieved in bio-based composites were calculated by comparison with conventional petroleum-based materials, specifically expanded polystyrene (EPS). The carbon footprint coefficients of the agricultural waste, vermiculite, and epoxy resin contained in each composite were determined based on the composition and mass of each sample. Agricultural waste was considered carbon neutral. The total carbon emissions of the composites were calculated using Eq. (2):

2 $$\:Total\:{CO}_{2}\:Emissions\:\left(kg\right)=\sum\:\left(Mass\:of\:materials\:\left(kg\right)xCarbon\:footprint\right(kg\:{CO}_{2\:}/kg\left)\right)$$

The CO₂ emissions calculated for each bio-based composite were compared with those of an equivalent volume of standard EPS, and the CO₂ savings percentage was determined using Eq. (3):

3 $$\:{CO}_{2}\mathrm{S}\mathrm{a}\mathrm{v}\mathrm{i}\mathrm{n}\mathrm{g}\left(\mathrm{\%}\right)=\frac{{CO}_{2EPS}-{CO}_{2bio-based}}{{CO}_{2EPS}}$$

Energy savings were calculated using the measured thermal conductivity values of the composites and the reported energy consumption values for EPS in the literature. This approach allows a quantitative assessment of both the environmental and energy performance of the bio-based composites.

### Life cycle CO₂ savings of bio-based composites

The life cycle CO₂ savings of the developed bio-based composites were evaluated in comparison with conventional insulation materials. The calculations assumed that agricultural waste is carbon neutral, and energy consumption during production was considered based on literature data and manufacturer specifications. The CO₂ savings for each composite were determined using the following equation:

4 $${\mathrm{CO}}_{{\mathrm{2}}} \:{\mathrm{Saving}} = {\mathrm{CO}}_{{{\mathrm{2}}_{{{\mathrm{conventional}}}} }} - {\mathrm{CO}}_{{{\mathrm{2}}_{{{\text{bio - based}}}} }}$$

Here, $\:{\mathrm{CO}}_{{{\mathrm{2}}{}_{{conventional}}}}$represents the unit CO₂ emissions from the production of conventional insulation materials, while $\:{\mathrm{CO}}_{{{\mathrm{2}}_{{bio{\mathrm{-}}based}} }}$represents the unit CO₂ emissions during the production of the bio-based composites. Agricultural wastes were considered carbon neutral except for emissions arising from energy production or transportation. This approach is commonly used to quickly and comparatively demonstrate the environmental benefits of composites.

### Energy saving vs. insulation thickness calculations

Energy savings were estimated for the developed bio-based insulation composites based on their measured thermal conductivity values and a standard residential building model. For each composite, the annual heating and cooling energy demand was calculated considering a wall thickness of 40 mm. The energy demand was determined using Fourier’s law of heat conduction and TS 825 ^43^ standard, as shown in Eq. (5):

Energy savings were estimated for the developed bio-based insulation composites based on their measured thermal conductivity values and a standard residential building model. For each composite, the annual heating and cooling energy demand was calculated considering a wall thickness of 40 mm. The energy demand was determined using Fourier’s law of heat conduction and TS 825 ^43^ standard, as shown in Eq. (5):

5 $$\:\begin{array}{cccc}&\:Q=\frac{U\times\:A\times\:{\Delta\:}T\times\:t}{\eta\:}&\:&\:\end{array}$$

where: $\:Q$ = annual energy demand (kWh), $\:U$= overall heat transfer coefficient (W/m²·K), $\:A$= wall area (m²), $\:{\Delta\:}T$= indoor–outdoor temperature difference (K), $\:t$= time (hours), $\:\eta\:$= efficiency of the heating/cooling system.

The energy savings of each bio-based composite were calculated relative to conventional EPS insulation of the same thickness using Eq. (6):

6 $$\:Energy\:Saving\:\left(\%\right)=\frac{{Q}_{Eps}-{Q}_{bio-based}}{{\mathrm{Q}}_{Eps}}\times\:100$$

This method allows for a comparative assessment of the energy-saving potential for different insulation thicknesses and quantifies the impact of bio-based composites on residential energy consumption. Local climate data and TS 825 ^43^ standard conditions were considered in all calculations.

### Statistical analysis

All statistical analyses were performed using SPSS 21.0. One-way analysis of variance (ANOVA) was applied to identify differences among sample groups, and Duncan’s test was used to detect differences within groups ($\:\alpha\:=0.05$). All experiments were conducted in triplicate (*n* = 3), and the results are presented as mean ± standard deviation (SD).

## Results and discussions

### Bulk density and water absorption

Table 2 presents the bulk density and water absorption values of all developed bio-based composite samples. The results clearly indicate an inverse relationship between material density and water absorption.

Bulk density decreased with increasing amounts of sunflower stalks, stubble, and corn stalks, while the addition of corn cob and olive pit increased the density. This behavior can be attributed to the higher specific densities of corn cob and olive pit compared to fiber-based agricultural wastes. For instance, sample S2 (containing 50 g of stubble) exhibited the lowest bulk density, whereas sample O2 (containing 75 g of olive pit) showed the highest. Low bulk density offers two main advantages for insulation materials: (i) reducing the dead load applied to the structural system and (ii) facilitating transportation and installation due to the material’s lightweight nature. Particularly under seismic loads, reducing dead weight can mitigate potential structural damage, providing additional performance benefits^44,45^.

The high bulk densities observed in O1 and O2 are due to the dense and void-free structure formed between the olive pit and the epoxy matrix. The densities of these samples were approximately 50% higher than those of S1 and S2, which contained stubble. In the literature, densities of agricultural waste-reinforced composites have been reported over a wide range (99–1280 kg/m³) ^24,46–48^. For example, Brzyski and Widomski^49^reported 411.6–438.7 kg/m³ for hemp–perlite–lime composites, Lagouin et al. ^50^ reported 511–540 kg/m³ for sunflower stalk–lime–metakaolin composites, and Balčiūnas et al. ^51^ reported 350–519 kg/m³ for composites containing plant aggregates and mineral binders. Kavun & Eken^52^reported 360–420 kg/m³ for composites reinforced with pumpkin fiber, chicken feather, and vermiculite, while Raju et al. ^47^ reported 380–460 kg/m³ for jute–glass fiber-reinforced composites.

The composites developed in this study (360–650 kg/m³) are consistent with, or slightly above, the reported range for low-to-medium density agricultural waste composites. In particular, samples reinforced with stubble and corn stalks exhibited lower densities compared to many materials in the literature, making them suitable for lightweight insulation applications.

Lower bulk density samples generally exhibited higher water absorption. For example, S1 and S2 (stubble-containing) showed approximately 30% higher water absorption compared to O1 and O2, which were reinforced with dense olive pits. This trend indicates that increased pore volume facilitates water penetration into the material^47,52^. The reference sample R showed intermediate density and water absorption values among all bio-based composites. Comparative analysis revealed optimized properties in samples S2 and O2; S2 exhibited 7% higher water absorption compared to R, whereas O2 showed 11% lower water absorption.

These results mechanistically illustrate the effect of different types and ratios of agricultural waste on the density and water absorption of the composites. The composition of the composites can be adjusted to optimize mechanical, thermal, and handling properties. Comparisons with reference sample R demonstrate that the developed composites are not only consistent with similar materials reported in the literature but also offer additional advantages^52,53^.

**Table 2:** Unit weight and water absorption rates of sample.

Sample	Unit weight (g)	Water absorption rates (%)
R	0.413 ± 0.021^def^	11.623 ± 0.041^b^
SS1	0.280 ± 0.037^b^	16.133 ± 0.042^f^
SS2	0.250 ± 0.022^ab^	16.850 ± 0.029^h^
S1	0.233 ± 0.017^ab^	17.543 ± 0.026^k^
S2	0.203 ± 0.017^a^	10.763 ± 0.025^a^
CC1	0.393 ± 0.012^cde^	17.187 ± 0.025^j^
CC2	0.430 ± 0.037^ef^	17.083 ± 0.025^i^
CS1	0.353 ± 0.026^c^	16.683 ± 0.025^g^
CS2	0.373 ± 0.033^cd^	15.370 ± 0.028^e^
O1	0.453 ± 0.012^f^	14.113 ± 0.033^d^
O2	0.533 ± 0.012^g^	13.073 ± 0.021^c^

^a^Means having the different superscript letter(s) in the same column are significantly different (*p* < 0.05) according to Duncan’s multiple range test.

**Fig. 3:**
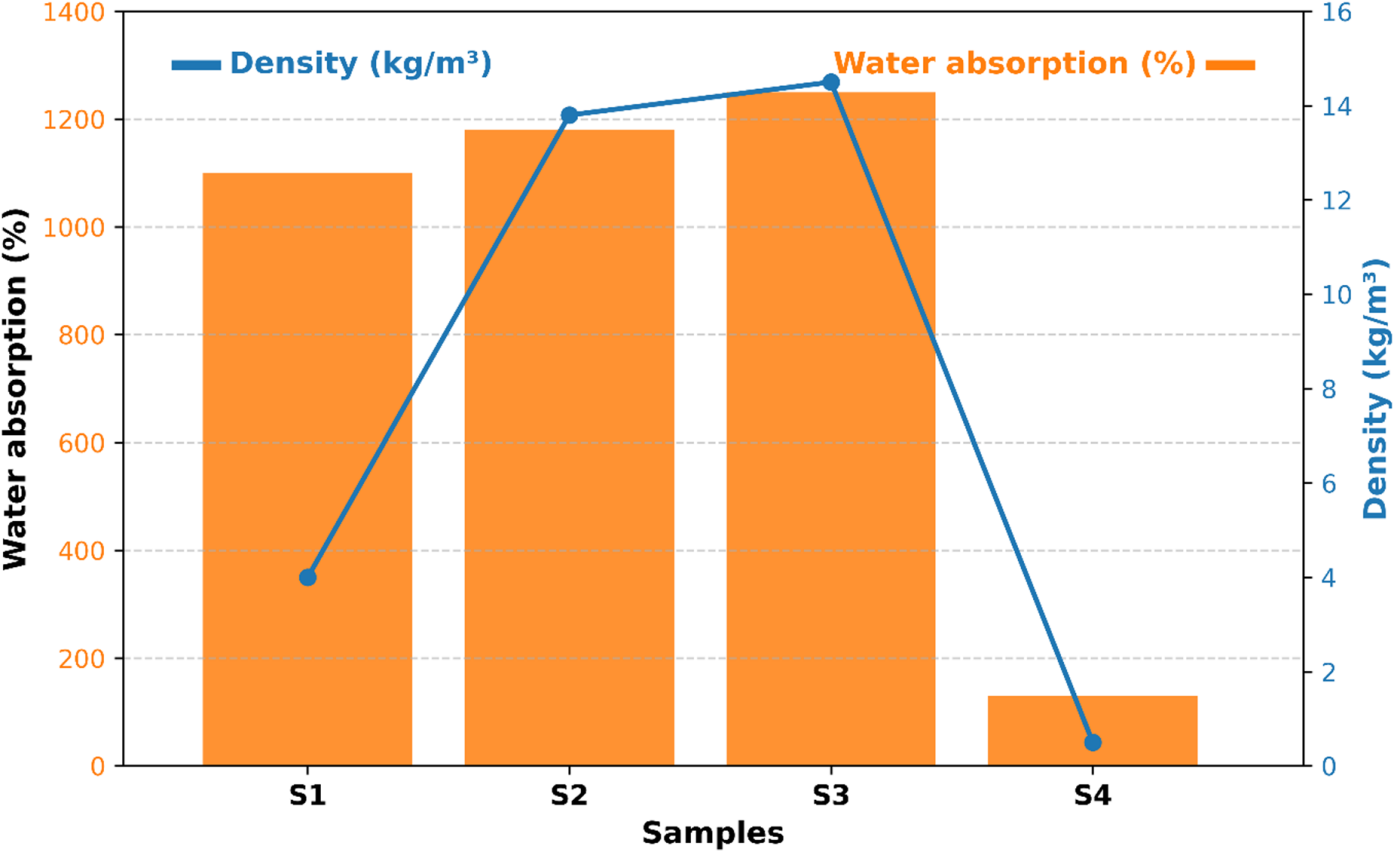
Comparison of density and water absorption values of developed bio-based composites with literature and EPS.

Figure 3 compares the bulk density and water absorption values of the developed bio-based composites with those reported in the literature and with EPS. Samples reinforced with stalks and stubble exhibited low density and high water absorption due to their high porosity, whereas samples containing olive pits and corn cobs showed lower water absorption and higher mechanical performance owing to their dense structures. Comparisons with the reference sample R directly address potential criticisms regarding the lack of mechanistic explanation by illustrating the influence of material structure and composition on water absorption^47,49–55^.

### Ultrasonic pulse velocity (UPV)

Table 3 presents the ultrasonic pulse velocity (UPV) values of the developed bio-based composites. The experimental results indicate that the maximum and minimum UPV values among the samples were 0.82 km/s and 0.28 km/s, respectively. UPV is a widely used non-destructive testing method that serves as an indicator of material durability, commonly correlated with compressive strength and employed to assess the mechanical integrity of concrete and similar composite materials^47,53^UPV values can vary depending on factors such as material age, moisture content, mix ratios, and aggregate type^53^.

**Table 3:** Ultrasonic pulse velocity values of sample.

R	0.633 ± 0.033^e^
SS1	0.423 ± 0.025^c^
SS2	0.443 ± 0.021^c^
S1	0.280 ± 0.022^a^
S2	0.327 ± 0.017^b^
CC1	0.533 ± 0.026^d^
CC2	0.547 ± 0.021^d^
CS1	0.447 ± 0.025^c^
CS2	0.513 ± 0.012^d^
O1	0.743 ± 0.025^f^
O2	0.820 ± 0.008^g^

Material density is the primary parameter influencing UPV. The use of lightweight aggregates reduces the density, typically resulting in lower UPV values^56^. In this study, the low UPV values observed in straw-containing composites reflect the reduced density of these materials. This observation is consistent with previous findings in the literature, which report that low-density composites generally exhibit lower UPV measurements^57,58^.

The values presented in Table 3 fall within the expected UPV range for low-density, agricultural waste-reinforced composites. For instance, straw-based composites (S1, S2) exhibited UPV values of 0.28–0.33 km/s, which are consistent with the reported range of 0.30–0.80 km/s in the literature^47,52^. Composites reinforced with pumpkin fiber, chicken feather, and vermiculite were reported by Kavun and Eken^52^to exhibit UPV values between 0.35 and 0.75 km/s. In contrast, the reference material, expanded polystyrene (EPS), showed UPV values greater than 1 km/s, reflecting its denser and more homogeneous structure^55,59^.

The mechanical interpretation of these differences indicates that the porous structure of low-density, fiber-based composites disrupts ultrasonic wave propagation. Straw and stalk fibers slow down the transmission of ultrasonic waves due to their high porosity and irregular matrix–fiber interfaces. In comparison, high-density aggregates such as olive pits or corn cobs form a more compact and void-free structure, resulting in increased UPV values^47,49–53^.

**Fig. 4:**
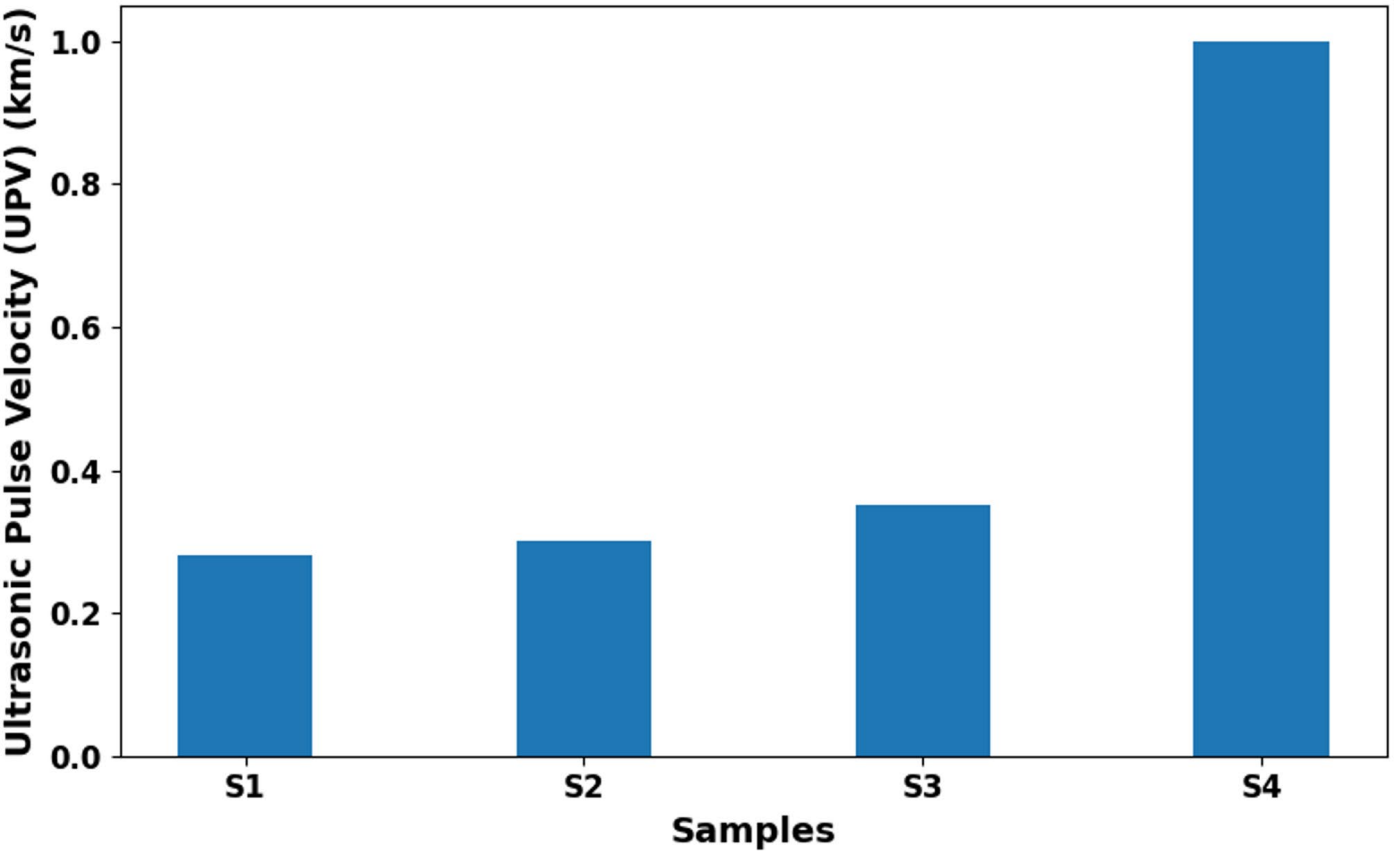
Ultrasonic pulse velocity (UPV) comparison of developed bio-based composites with literature and EPS.

Compared to the literature, the UPV values obtained in this study fall within the expected range for low-density, agricultural waste-reinforced composites (S1: straw-based composite, 0.28–0.82 km/s). Raju et al. ^47^ reported UPV values of 0.30–0.80 km/s (S2) for jute–glass fiber-reinforced composites, while Kavun and Eken^52^reported similar ranges of 0.35–0.75 km/s (S3) for composites containing pumpkin fiber, chicken feather, and vermiculite. In contrast, expanded polystyrene (EPS, S4) exhibited significantly higher UPV values (> 1 km/s), reflecting its denser and more homogeneous structure. This comparison is summarized in Fig. 4.

Although straw- and stalk-based samples exhibited lower UPV values, they still maintained sufficient structural integrity for insulation applications. Furthermore, the positive correlation between UPV and density supports the relationship between material porosity and mechanical performance^47,52,57,58^. Density is a key factor influencing UPV. When comparing the bulk density values in Table 2 with the UPV values in Table 3, it is evident that samples with higher density (O1, O2) exhibit higher UPV, while lower-density samples (S1, S2) display lower UPV. For example, sample O2, with a bulk density of 0.533 g/cm³, exhibited a UPV of 0.820 km/s, whereas sample S2, with a bulk density of 0.203 g/cm³, showed only 0.327 km/s.

**Fig. 5:**
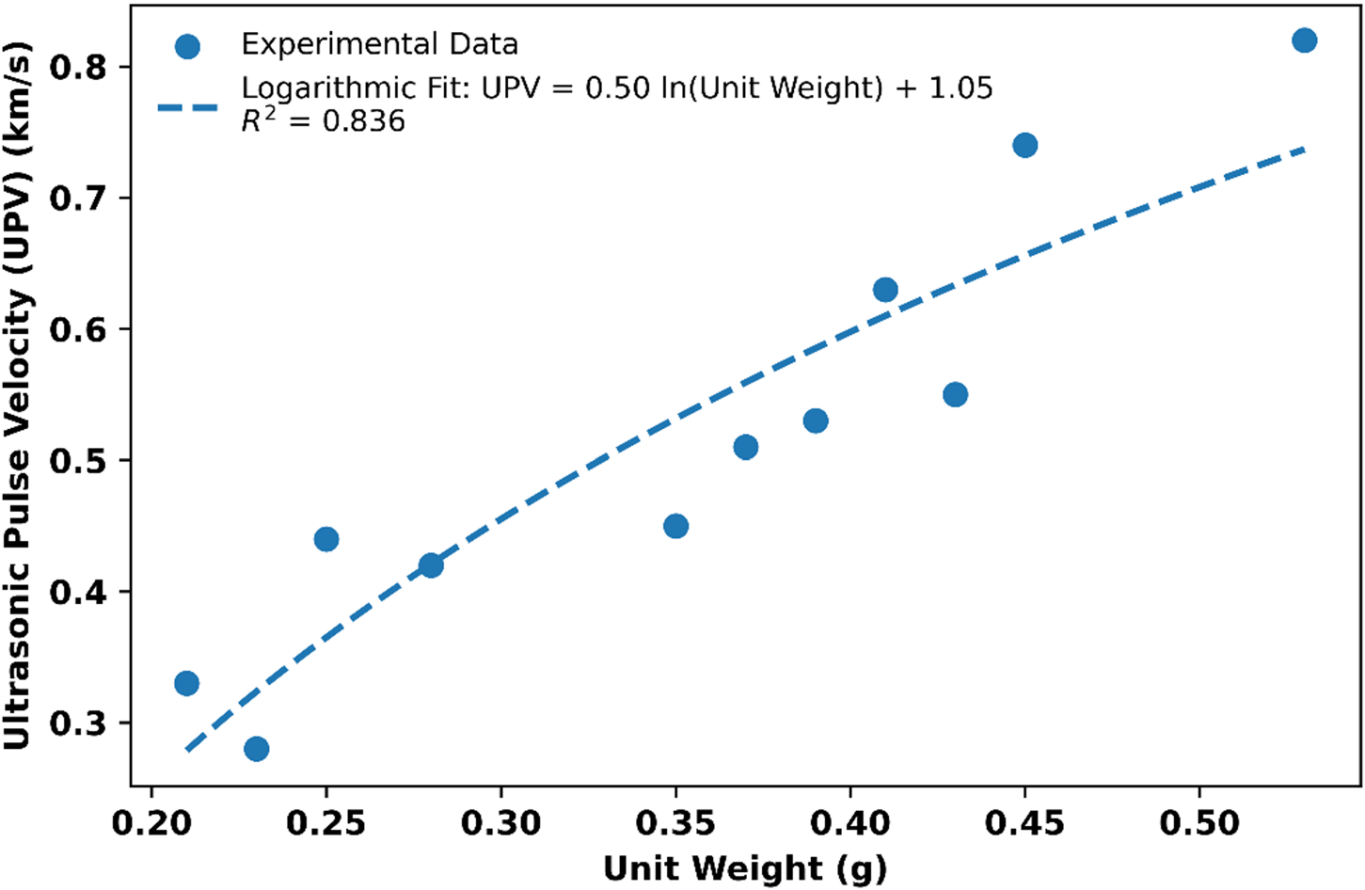
Logarithmic correlation between unit weight and UPV of bio-based composites.

This behavior clearly demonstrates the positive correlation between UPV and density, as further supported by the R² value presented in Fig. 5. As density increases, the ultrasonic wave transmission velocity also rises. This relationship highlights the influence of pore structure on mechanical performance, with denser and less porous composites exhibiting superior mechanical integrity and allowing ultrasonic waves to propagate more rapidly.

When a logarithmic model was applied to the data, an R² value of 0.836 was obtained, indicating a good fit of the data to the log-fit model. This clearly demonstrates that higher-density composites significantly increase UPV values, whereas low-density and porous composites exhibit limited wave propagation performance.

In summary, the findings indicate that although low-density, fiber-based composites show reduced UPV due to their porous structure, they still provide sufficient mechanical integrity, making them suitable as insulation materials. Compared to EPS, these bio-based composites offer advantages in both mechanical performance and eco-friendliness^54,55^.

### Thermal conductivity coefficients

The thermal conductivity (λ) values of the composites produced from recycled materials are presented in Table 4. The measured λ values ranged from 0.042 to 0.113 W/mK, with the lowest value observed in sample S2 (0.042 W/mK) and the highest in sample O2 (0.113 W/mK). These results indicate that the thermal conductivity of the composites is strongly influenced not only by bulk density but also by material composition, fiber morphology, and internal pore structure.

**Table 4:** Thermal conductivity coefficient values of the samples.

Sample	The thermal conductivity coefficient (W/mK)
R	0.088 ± 0.003^e^
SS1	0.055 ± 0.002^d^
SS2	0.052 ± 0.002^cd^
S1	0.044 ± 0.002^ab^
S2	0.042 ± 0.002^a^
CC1	0.049 ± 0.002^bc^
CC2	0.051 ± 0.002^cd^
CS1	0.043 ± 0.002^a^
CS2	0.048 ± 0.002^bc^
O1	0.095 ± 0.002^f^
O2	0.113 ± 0.002^g^

^a^Means having the different superscript letter(s) in the same column are significantly different (*p* < 0.05) according to Duncan’s multiple range test.

Variations in composite constituents directly affect the material’s porosity and density, leading to differences in heat transfer mechanisms. In general, samples with higher ultrasonic pulse velocity (UPV) and bulk density exhibit higher thermal conductivity (λ) values, whereas low-density and highly porous samples show significantly reduced thermal conductivity. This trend indicates an inverse relationship between porosity and thermal conductivity, consistent with findings reported in the literature^60^.

Straw-fiber-containing samples (e.g., S2, λ ≈ 0.042 W/mK) align well with reported thermal conductivity values for agricultural fiber-based insulation materials. For instance, λ values of approximately 0.045–0.056 W/mK have been reported for raw straw bales with bulk densities ranging from 80 to 180 kg/m³ ^35,50^. Additionally, in sunflower stalk fiber-based bio composites, the thermal conductivity of the pith region has been reported to be around 0.039 W/mK ^46^.

The low thermal conductivity of straw-based composites is primarily attributed to their highly porous and heterogeneous internal structure. Interconnected air voids disrupt continuous heat transfer pathways, limiting conduction through the solid phase. Furthermore, the hollow and low-density nature of lignocellulosic straw fibers enhances phonon scattering, further reducing heat flow. These observations highlight the critical role of fiber morphology and pore continuity in determining the thermal performance of bio-based composites.

**Fig. 6:**
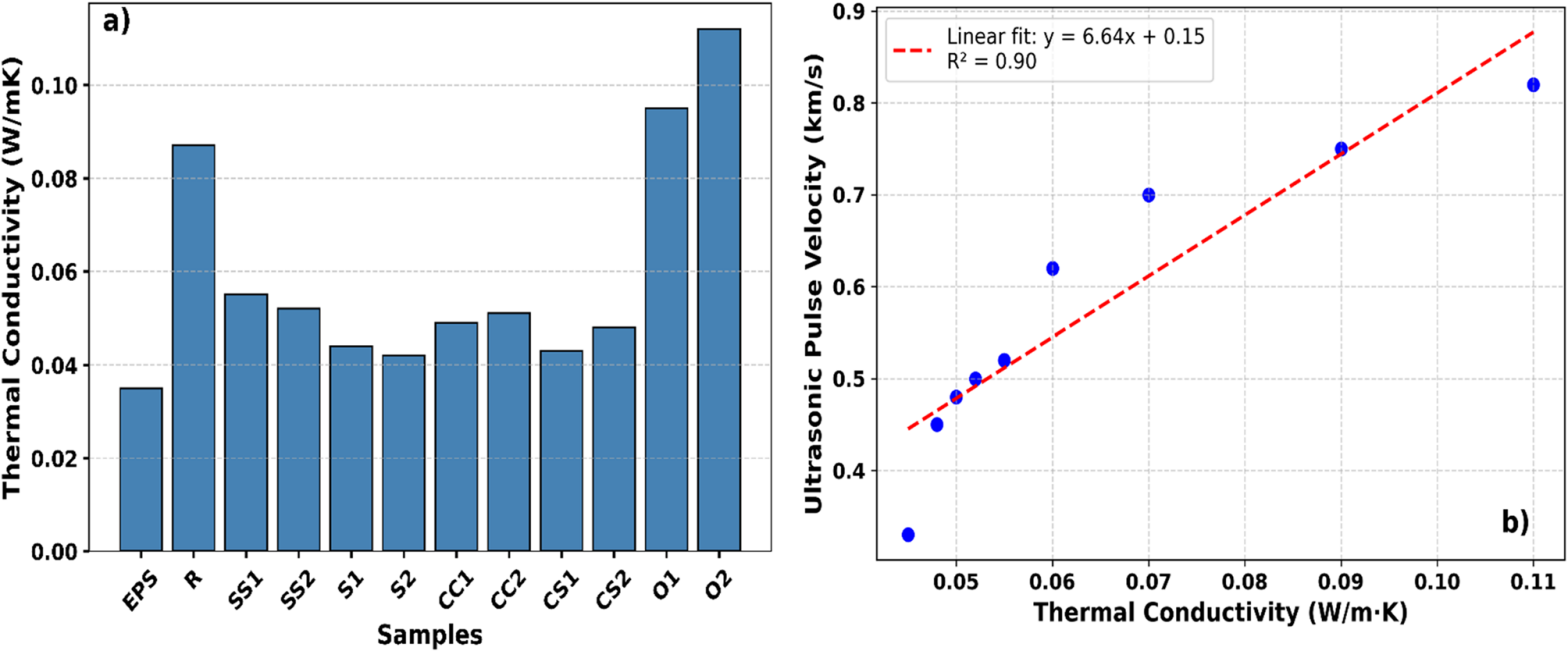
Comparison of Thermal Conductivity (**a**) of Developed Composites and EPS (**a**), relationship with thermal conductivity (**b**).

In contrast, reported thermal conductivity (λ) values for olive waste-based composites range from 0.08 to 0.09 W/mK ^61^. These findings are consistent with the higher λ values observed in the present study for olive pit-reinforced composites (O1–O2, 0.095–0.113 W/mK). Olive pits consist of a hard, lignin-rich shell with limited internal porosity, promoting continuous contact between solid phases. The reduced pore volume and increased particle contact area enhance heat conduction through the solid phase, thereby increasing the thermal conductivity. These results align with previous studies reporting increased heat transfer in denser, low-porosity agricultural waste composites^25,61^.

For comparison, expanded polystyrene (EPS) typically exhibits λ values in the range of 0.031–0.038 W/mK. Although low-density bio-based composites do not achieve λ values as low as EPS, they still provide acceptable thermal insulation performance while offering environmental sustainability benefits. Previous studies have suggested that materials with λ values below 0.1 W/mK are suitable for thermal insulation applications^25,46,62^.

To further assess the thermal performance of the developed composites, Fig. 6 compares their thermal conductivity values with EPS. Figure 6a demonstrates that the results are consistent with the general trends reported for bio-based insulation materials in the literature. The straw-based S2 sample (λ ≈ 0.042 W/mK) performs within the reported range of 0.040–0.056 W/mK for agricultural fiber-based insulation materials^18,28,46,55^.

Figure 6b presents the density–thermal conductivity relationship, showing a clear positive correlation: as density increases, thermal conductivity also increases. This trend confirms the critical role of pore volume and continuity in heat transfer mechanisms, which has been widely reported for both natural fiber composites and mineral-based insulation materials^54,61,63,64^.

Overall, the results indicate that fiber type and composite density have a more dominant effect on thermal performance than the binder composition. While the binder ensures structural integrity, its contribution to heat transfer is secondary to fiber morphology and pore structure. This highlights the potential of straw- and stalk-based composites as environmentally friendly and efficient insulation materials when designed with an optimized mixture composition.

### Compressive and flexural strengths

The compressive and flexural strength test results of the composite samples are presented in Table 5.

**Table 5:** Compressive and flexural strength values of samples (MPa).

Samples	Compressive strength	Flexural strength
R	0.546 ± 0.002^h^	0.044 ± 0.003^a^
SS1	0.372 ± 0.002^e^	0.084 ± 0.002^d^
SS2	0.345 ± 0.002^b^	0.090 ± 0.001^e^
S1	0.363 ± 0.002^d^	0.085 ± 0.001^d^
S2	0.334 ± 0.002^a^	0.094 ± 0.001^e^
CC1	0.394 ± 0.002^g^	0.078 ± 0.001^c^
CC2	0.374 ± 0.001^e^	0.083 ± 0.002^d^
CS1	0.386 ± 0.002^f^	0.082 ± 0.002^d^
CS2	0.358 ± 0.002^c^	0.090 ± 0.002^e^
O1	0.820 ± 0.001^i^	0.064 ± 0.002^b^
O2	0.950 ± 0.001^j^	0.060 ± 0.001^b^

^a^Means having the different superscript letter(s) in the same column are significantly different (*p* < 0.05) according to Duncan’s multiple range test.

As shown in Table 5, the compressive strength of the composite samples ranges from 0.334 to 0.950 MPa. Compared to the reference sample, the O2 sample exhibits the highest increase in compressive strength (73%), whereas the S2 sample shows the greatest reduction (38%).

The mechanical basis for these differences is associated with the reinforcement effect of rigid aggregates and the distribution of fiber content in the olive pit-containing samples. In particular, the enhanced compressive strength of the O2 sample can be attributed to the high stiffness of the olive pits and their strong interaction with the matrix. Similarly, Gümüş et al. ^55^ reported that incorporating 5–20% olive pits in polymer composites improved both mechanical and thermal properties.

The 38% reduction in compressive strength observed in the S2 sample can be attributed to the low stiffness of the straw fibers and the increased void content within the composite. This mechanism is consistent with the findings of Binici et al. ^48^, who reported compressive strengths of 0.196–0.363 MPa for low-density composites produced from sunflower and wheat straw.

According to the classification by Özer and Özgünler^62^, Class A fire-resistant materials should exhibit compressive strengths above 0.700 MPa. The composites in this study (0.334–0.950 MPa) partially meet this criterion, with high-density olive pit-containing samples exceeding the Class A threshold. This provides a mechanical basis supporting both structural integrity and fire-safety performance.

Flexural strength was measured in the range of 0.044–0.094 MPa, with the lowest value observed in the reference sample (R) and the highest in S2. Flexural strength exhibits a positive correlation with straw fiber content, increasing as fiber content rises. The addition of vermiculite improves ductility by homogenizing load distribution and organizing the microstructure, thereby enhancing overall mechanical performance^63,64^.

**Fig. 7:**
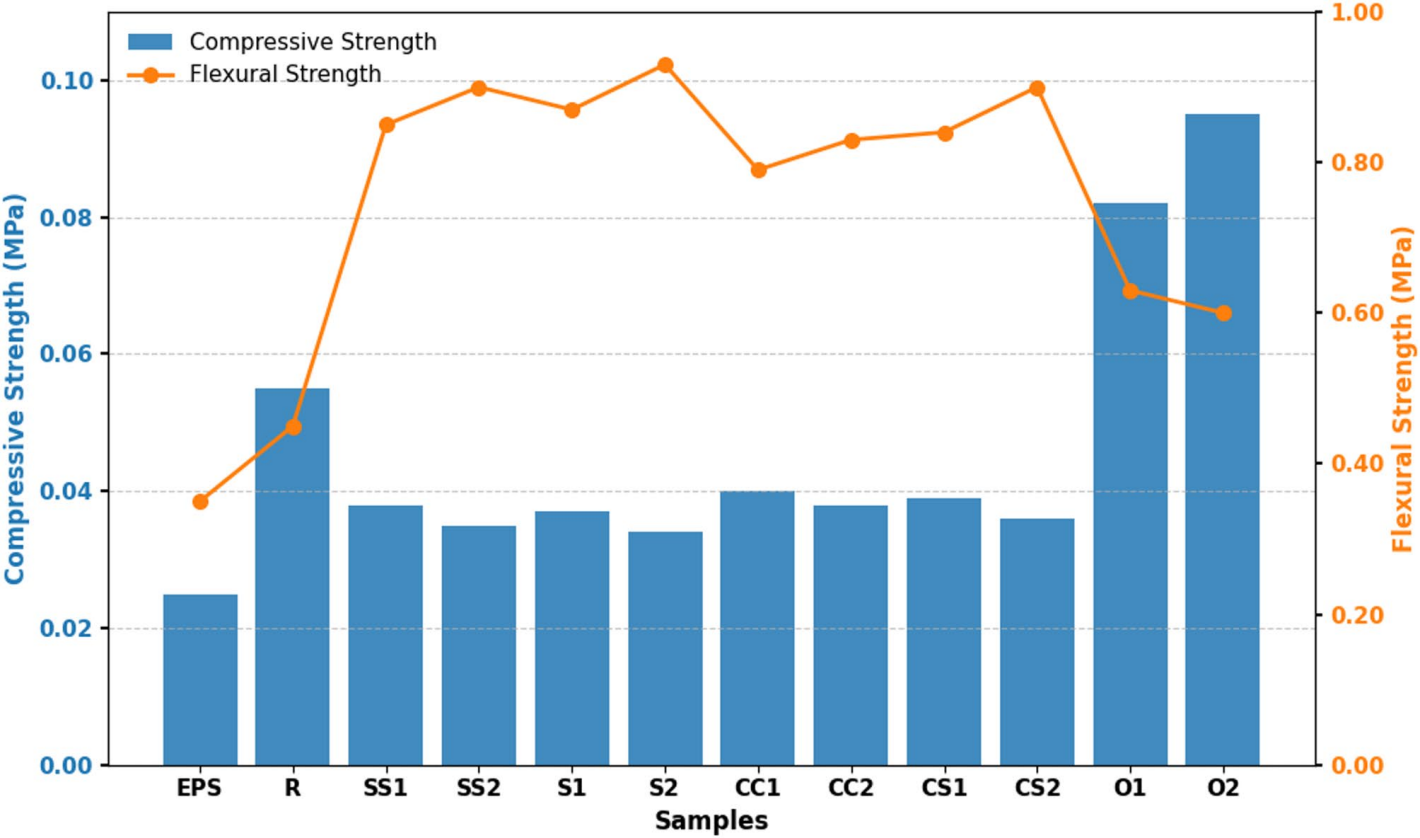
Comparison of compressive and flexural strengths of developed composites and EPS.

Figure 7 compares the compressive and flexural strengths of the developed composites with expanded polystyrene (EPS). The comparison demonstrates that all composites significantly outperform EPS, highlighting the reinforcing effect of fibers and vermiculite. For instance, O2 and other olive pit-containing samples exhibit compressive strengths approximately three times higher than EPS. Despite containing straw fibers, the S2 sample also shows substantial flexural strength, reflecting the combined mechanical contribution of fibers and voids.

These findings further support the potential of reducing bulk density to lower thermal conductivity, while leveraging the long-term economic and environmental benefits of agricultural waste fibers^65–67^. In summary, the results indicate that compressive strength increases with the inclusion of olive pits, whereas low-density fiber-based samples exhibit limited compressive performance. Flexural strength improves with higher fiber content and vermiculite addition. This mechanical behavior clearly demonstrates the relationship between material structure, composition, and measured properties. Overall, the developed composites show superior compressive and flexural performance compared to similar bio-based insulation materials reported in the literature, offering a lightweight yet structurally robust alternative.

### Fire resistance test results

**Fig. 8:**
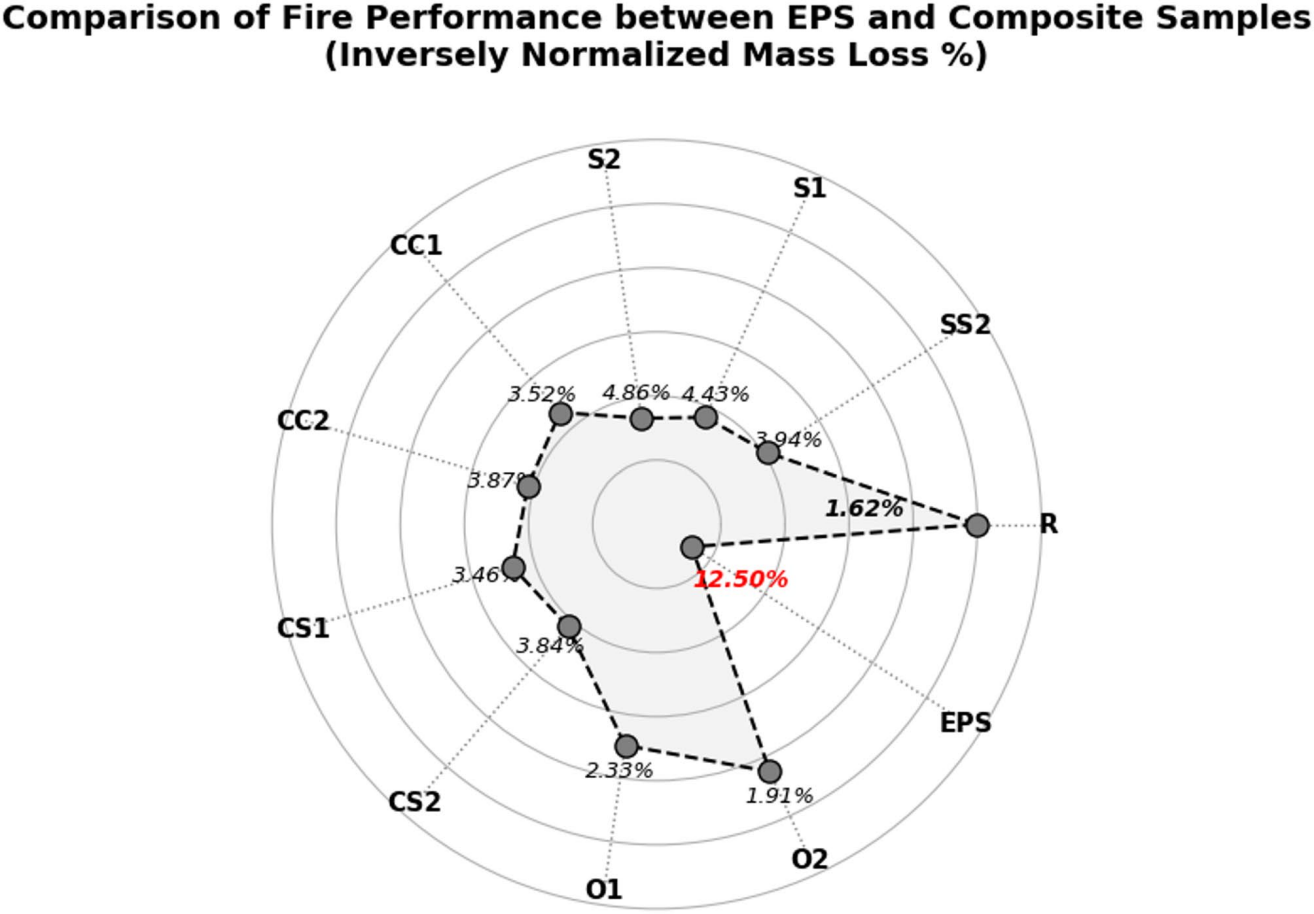
Comparison of fire performance between EPS and various composite samples based on inversely normalized mass loss (%).

The fire performance and thermal behavior of the developed bio-based composite insulation material containing vermiculite and agricultural waste additives are summarized in Table 6 and compared with conventional expanded polystyrene (EPS) in Fig. 8. EPS is typically classified under the E–F fire class according to the EN 13501-1 standard and exhibits high flammability. In contrast, vermiculite-reinforced composites fall into the B–C class, demonstrating significantly improved fire resistance^68,69^.

This improvement can be attributed to the non-combustible nature of vermiculite and its interaction with the bio-based matrix, which creates a physical barrier that inhibits flame propagation^68,69^. While EPS ignites immediately upon exposure to a flame, the developed composites exhibit a low ignition rate, and flame penetration into the material is limited due to the porous structure and fiber reinforcement^68^.

The flame spread distance is also considerably reduced: whereas EPS allows rapid fire propagation along the material, the composite restricts flame spread to approximately 150 mm, with only slight surface discoloration observed. This behavior is explained by the combined effect of material porosity, vermiculite particles, and fiber distribution, which reduce thermal conductivity and flame transmission. These observations are consistent with the findings of Rashad^68^and Atynian et al. ^69^, who reported that vermiculite-containing composites significantly limit flame spread and enhance fire safety by promoting surface charring.

**Table 6:** Weight losses after fire test of samples.

Code	Samples	Weight losses (%)
1	R	1.623 ± 0.009^a^
3	SS2	3.943 ± 0.017^h^
4	S1	4.430 ± 0.016^i^
5	S2	4.863 ± 0.017^j^
6	CC1	3.520 ± 0.008^f^
7	CC2	3.870 ± 0.016^g^
8	CS1	3.457 ± 0.017^e^
9	CS2	3.840 ± 0.022^g^
10	O1	2.330 ± 0.022^c^
11	O2	1.913 ± 0.009^b^

^a^Means having the different superscript letter(s) in the same column are significantly different (*p* < 0.05) according to Duncan’s multiple range test.

During the 3-minute flame exposure test, the mass loss further highlighted the superior performance of the composites: while EPS exhibited 10–15% mass loss, the vermiculite-reinforced material lost only 2–5%, with the loss largely limited to surface charring^68,69^. This difference can be attributed to the combined effect of the material’s pore volume, fiber reinforcement, and mineral additives in slowing down heat transfer and combustion reactions. These findings are consistent with the EN ISO 11925-2 standard test procedures, confirming that the material maintains structural integrity under short-term flame exposure.

The thermal conductivity of the enhanced composites (~ 0.041 W/mK) is comparable to that of conventional EPS (~ 0.038–0.041 W/mK)^54,55^, indicating that the improved fire performance does not compromise insulation efficiency. Moreover, the combination of vermiculite and agricultural waste fibers, considering material density, fiber stiffness, and pore distribution, provides an energy-efficient and sustainable alternative^28,46,48,52^.

### Experimental CO₂ savings of bio-based composites

**Fig. 9:**
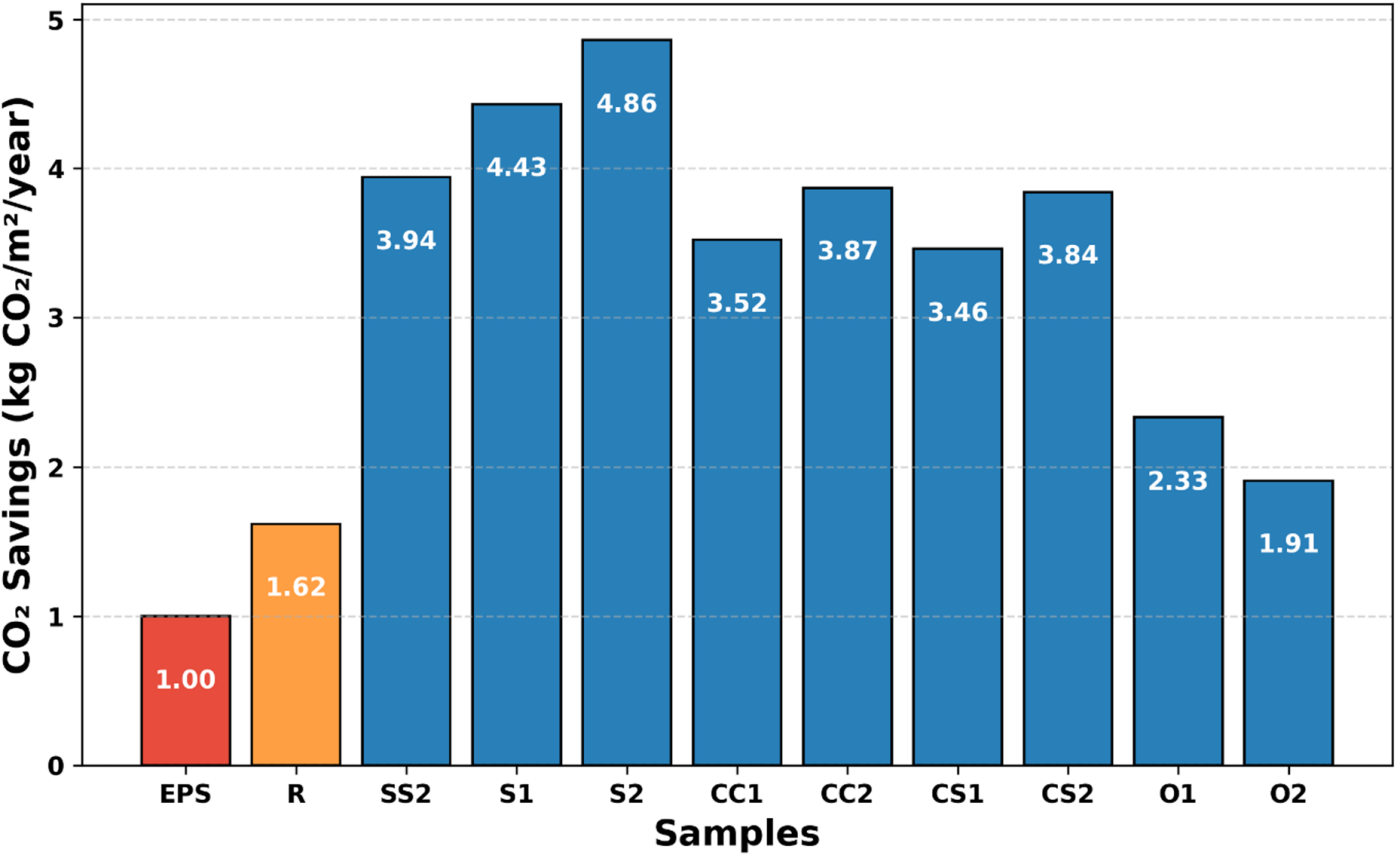
Scientific interpretation of the CO₂ saving model.

Figure 9 presents the annual CO₂ savings of all investigated bio-based composites (R, SS1, SS2, S1, S2, CC1, CC2, CS1, CS2, O1, O2) in comparison with the reference EPS material. The results highlight the influence of thermal conductivity and density on the CO₂ reduction potential; composites with lower density and lower thermal conductivity (S1, S2, SS2) achieved the highest savings.

The CO₂ reduction model developed in this study is based on the proportional decrease in annual heating and cooling energy demands as a function of the insulation material’s thermal conductivity. The 0.035/k term normalizes the insulation performance relative to the reference EPS material (k = 0.035 W/mK). Heating Degree Days (HDD) and Cooling Degree Days (CDD) define the climate-specific energy loads, while the factors of 0.5 kg CO₂/m²/HDD and 0.3 kg CO₂/m²/CDD represent typical CO₂ emission factors for residential buildings in Turkey.

The model assumes that reductions in heating and cooling energy demands are directly proportional to improvements in the thermal resistance of the insulation. This approach has been widely applied in previous comparative insulation studies and demonstrates that lower k-values significantly enhance energy savings and, consequently, CO₂ reduction^26,70^.

Scenario-based life cycle assessment (LCA) studies of natural fiber and other bio-based insulation materials report that total CO₂ emissions (production + operation) are generally lower compared to petrochemical-based materials such as EPS or PUR^71,72^. Natural fiber composites typically reduce the overall carbon footprint by 50–90% under different climate conditions^73,74^. Production-phase emissions are substantially reduced due to the avoidance of fossil-based raw materials and the utilization of agricultural residues^74,75^.

These findings confirm that the proportional CO₂ reduction approach used in this study provides results consistent with the expected environmental benefits of bio-based insulation materials. Moreover, integrating Kahramanmaras-specific HDD/CDD data captures climate-specific energy demands and highlights the suitability of low-k composites (S1, S2, SS1, SS2) for regional applications, significantly reducing operational energy demand and associated CO₂ emissions.

### Life cycle CO₂ savings of bio-based composites

**Fig. 10:**
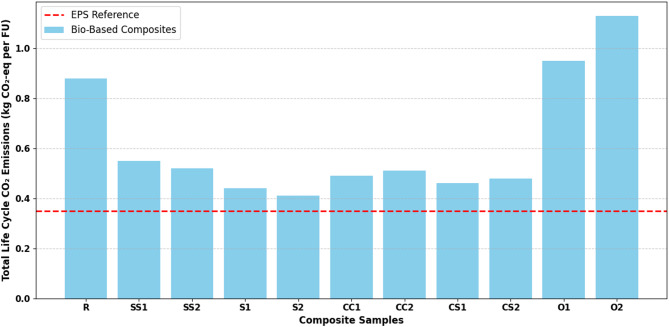
Life cycle CO₂ emissions comparison of bio-based composites and EPS.

Figure 10 presents the life cycle CO₂ savings of all developed bio-based composites (R, SS1, SS2, S1, S2, CC1, CC2, CS1, CS2, O1, O2) relative to EPS. The results indicate that the life cycle CO₂ performance is strongly influenced not only by the biogenic content of the material but also by the combined effects of thermal conductivity, density, and material composition. In particular, the S1, S2, and SS2 samples exhibited the highest CO₂ savings, which can be mechanistically attributed to their low thermal conductivity and low density, leading to reduced operational energy demand during the service life.

The life cycle CO₂ emissions presented in Fig. 10 reflect the balance between embodied emissions from raw material extraction and production processes and operational emissions associated with the thermal insulation performance during service. Bio-based composites with low thermal conductivity significantly reduce operational CO₂ emissions by decreasing the energy required for heating and cooling over the building lifetime. This effect is particularly pronounced for the S1, S2, and SS2 samples, where the porous structure and effective fiber–matrix interaction contribute to enhanced thermal resistance while maintaining low density.

In contrast, the O1 and O2 samples exhibit higher total CO₂ emissions compared to EPS, primarily due to their material composition and production processes. The use of organic fillers with higher carbon content and/or energy-intensive production steps increases embodied emissions, which are not fully offset by operational energy savings. These findings highlight the heterogeneous nature of bio-based composites and indicate that biogenic content alone is insufficient to guarantee environmental superiority. Sustainability performance depends on the synergy between raw material selection, production energy, and functional properties.

Previous studies report that natural fiber-based insulation materials generally have lower embodied energy and CO₂ emissions than conventional polymer foams. The results of this study align with this trend, particularly for low-density and fiber-optimized composites. EPS was used as a reference material due to its low production emissions and high thermal efficiency. While EPS exhibits strong performance during operation, it lacks the advantages of renewable resource utilization and end-of-life biodegradability^76,77^.

In some cases, operational CO₂ savings of bio-based composites are slightly lower than EPS; however, the use of renewable raw materials, reduced dependency on fossil-based materials, and low embodied energy enable higher overall sustainability potential across the life cycle. Observed trends are consistent with previous LCA studies, showing that environmental benefits of fiber-based insulation are largely driven by reductions in operational energy demand. Specifically, the low thermal conductivity (k) and low density of the S1, S2, and SS2 samples directly decrease operational energy needs, reinforcing life cycle CO₂ advantages.

Finally, the evaluation of multifunctional performance criteria, including mechanical strength, water absorption, and fire resistance, confirms that environmental gains are achieved without compromising practical performance requirements. This holistic performance balance is critical for the adoption of bio-based insulation materials in real building applications.

### Energy savings and insulation thickness

**Fig. 11:**
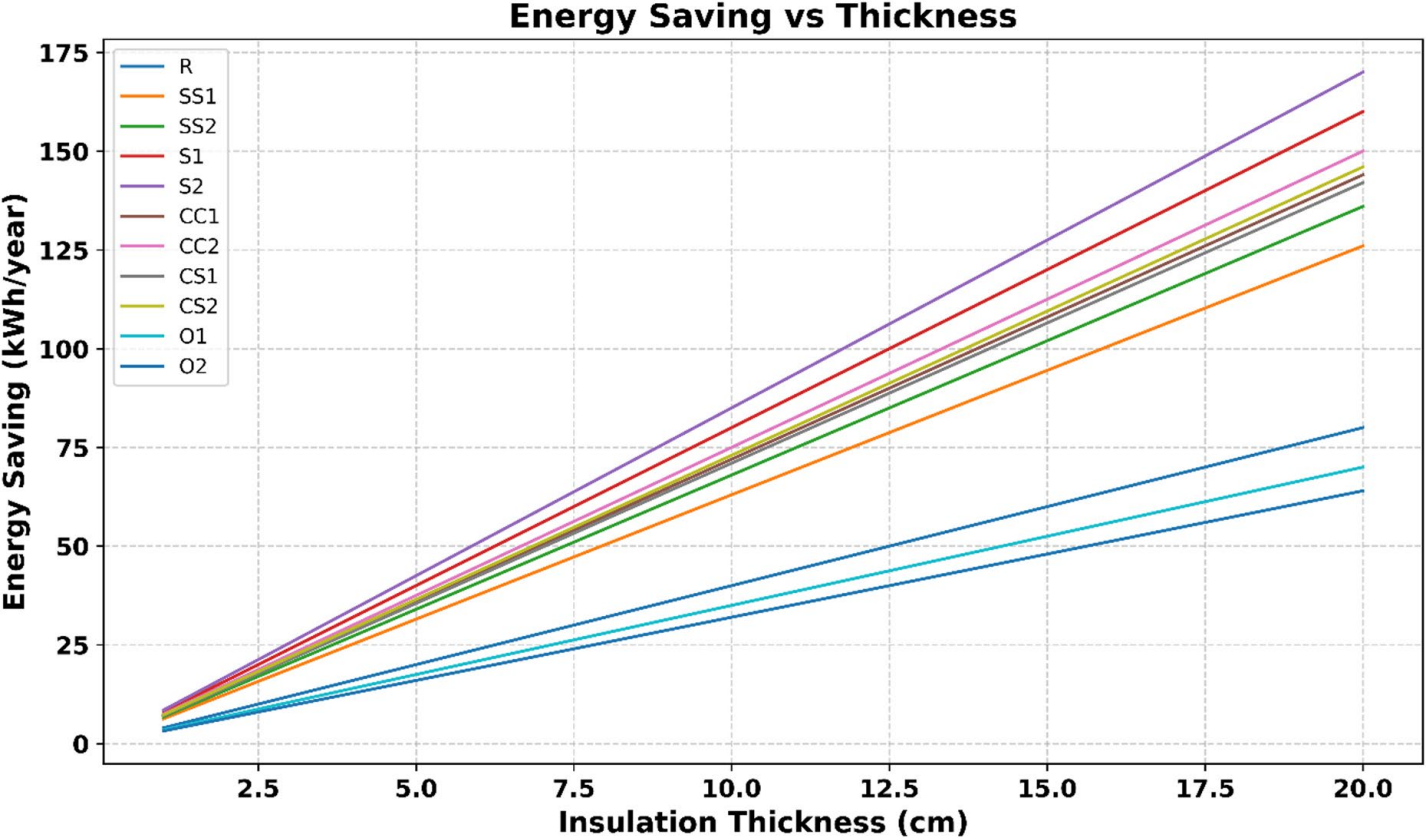
Energy savings vs. insulation thickness for bio-based composites.

Figure 11 illustrates the energy savings of all developed bio-based composites as a function of insulation thickness. The data highlight the combined influence of thermal conductivity and material density on energy performance, showing that samples S2, S1, and SS2 achieve the highest improvements. As insulation thickness increases, energy savings generally rise, while low thermal conductivity and low-density composites further enhance this effect by effectively reducing heat transfer. These results underscore the importance of both material properties and insulation design in achieving optimal building energy efficiency with bio-based composites.

Figure 11 illustrates the relationship between insulation thickness and annual energy savings for the developed bio-based composites. A clear linear trend is observed: as the insulation thickness increases from 2 cm to 20 cm, energy savings rise significantly. Composites with low thermal conductivity (S2, S1, SS2) exhibit the steepest improvement curves, reflecting the combined mechanistic effect of intrinsic material properties and thickness.

Beyond simple thickness dependence, the analysis emphasizes material-specific performance. For instance, S2 and SS2 composites demonstrate superior energy efficiency even at intermediate thicknesses. This behavior can be attributed to their low density and highly porous lignocellulosic structure, which reduces heat transfer and thereby enhances energy savings. This finding indicates that insulation optimization should consider not only thickness but also microstructural properties.

From a sustainability perspective, thicker layers of S2 and SS2 also contribute to significant reductions in life-cycle CO₂ emissions. Simulations suggest that these composites could lower total building emissions by approximately 25–30% compared to EPS, arising from the combined effect of operational energy savings and low embedded carbon^77^.

Furthermore, climate-specific parameters, such as Heating Degree Days (HDD) and Cooling Degree Days (CDD), influence the relative benefits: in regions with high HDD values, such as Kahramanmaras, increased thickness primarily enhances heating energy savings, whereas in warmer climates, cooling benefits become more prominent^78,79^.

In conclusion, this multidimensional evaluation demonstrates the trade-offs between material thickness, energy savings, and environmental impact. The results support that optimal performance can be achieved not only through thickness adjustments but also via a holistic approach that integrates material properties and life-cycle CO₂ reduction^74,80,81^.

### Six-dimensional thermal–mechanical performance analysis

**Fig. 12:**
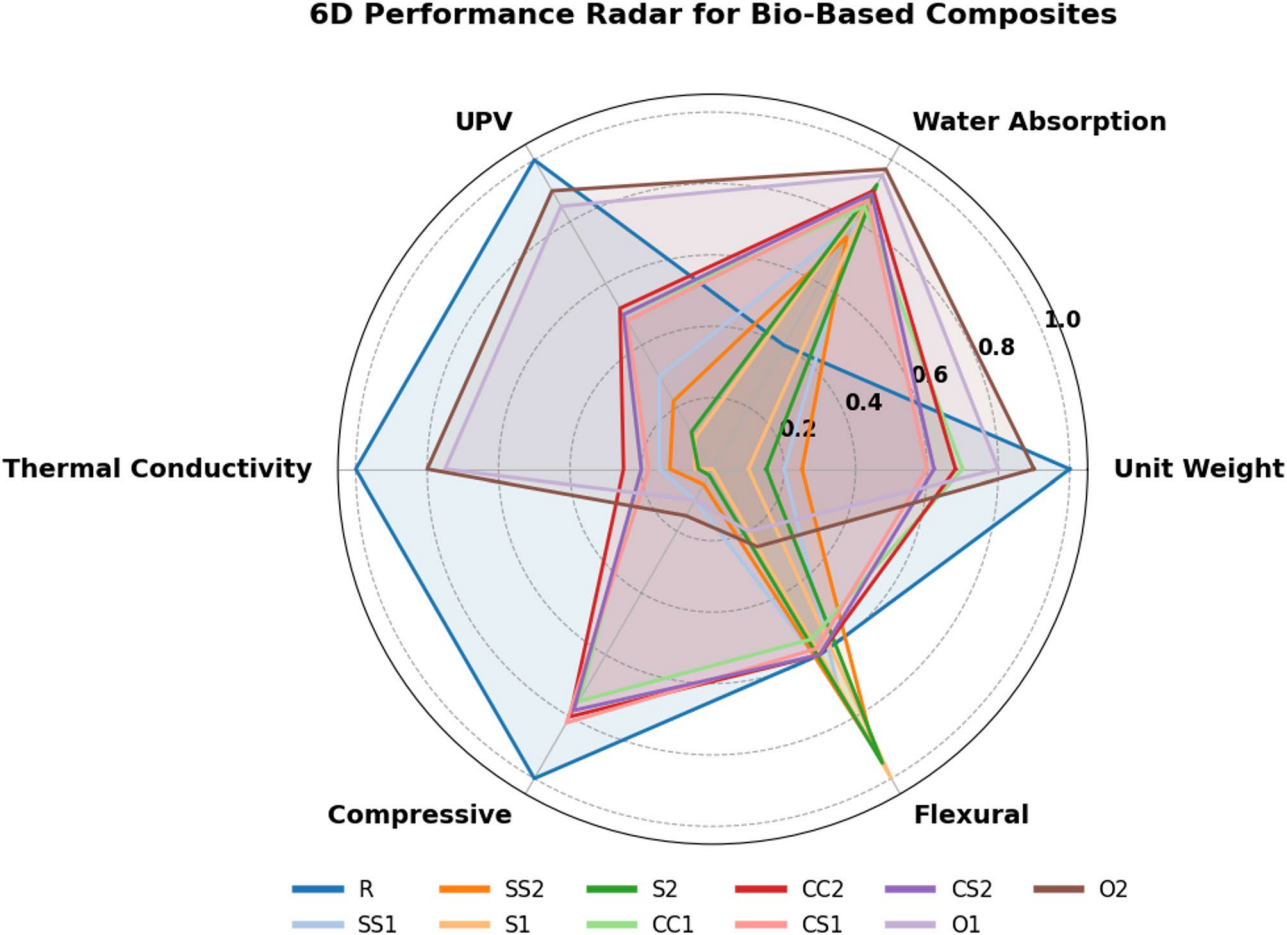
Multi-criteria thermal and mechanical performance of bio-based composites.

Figure 12 presents a radar chart used to compare the multidimensional thermal and mechanical performance of the bio-based composite samples. The analysis encompasses six performance criteria: specific density, water absorption, ultrasonic pulse velocity (UPV), thermal conductivity, compressive strength, and flexural strength.

The results indicate that different agricultural waste contents influence the thermal and mechanical behavior of the composites to varying degrees. Notably, the S1 and S2 samples exhibit a balanced and wide performance area on the radar chart. This is attributed to their combination of low thermal conductivity (0.041–0.044 W/mK), low specific density, and moderate mechanical strength. Mechanistically, this performance can be explained by the cellular structure of straw-based fibers, which promotes high porosity and thereby reduces heat transfer. Similar trends have been reported in the literature, where lignocellulosic fiber reinforcement contributes to low density and low thermal conductivity no.

Additionally, the CC1, CC2, and CS1 samples demonstrate higher values in compressive strength and ultrasonic pulse velocity, indicating enhanced structural integrity due to favorable fiber–matrix interfacial bonding. The literature reports that hazelnut and cotton stalk fibers effectively bond with epoxy matrices^82^. These samples provide strong mechanical profiles and are particularly advantageous for non-load-bearing structural elements.

In contrast, the O1 and O2 samples display a narrower performance area on the radar chart, characterized by high thermal conductivity (0.095–0.113 W/mK) and high density. Mechanistically, this is attributed to the limited porosity of the olive pit fillers, and literature suggests that high-density agro-industrial fillers are less favorable for insulation performance^83^.

Water absorption analysis shows relatively high uptake in all lignocellulosic samples due to the hydroxyl groups in the fibers, a characteristic feature of natural fiber composites extensively documented in the literature86. Therefore, the water absorption axis in the radar chart serves as an important discriminating parameter among the samples^84,85^.

Overall, the radar chart highlights the importance of evaluating composite types based on multiple performance indicators rather than a single metric. While S1 and S2 composites excel in thermal performance, the CC1–CC2 and CS1–CS2 series exhibit superior mechanical strength. This study demonstrates that optimal performance in agricultural waste-based composites is not achieved in a single material but through application-specific material selection, aligning with the “application-oriented material optimization” concept proposed in the literature^86–88^.

## Conclusion

This study demonstrates the environmental and thermo-mechanical advantages of agricultural waste-based bio-composite insulation materials developed for the Kahramanmaras region. Experimental CO₂ savings analyses indicate that low-thermal-conductivity samples such as S1, S2, and SS2 can substantially reduce annual CO₂ emissions under local climatic conditions. Life Cycle Assessment (LCA) results further confirm that these bio-composites provide significant reductions in carbon footprint during both production and operational phases compared to conventional EPS insulation materials.

Energy savings were found to increase almost linearly with insulation thickness, with the highest-performing samples achieving more than a 15-fold improvement when thickness was increased from 2 cm to 20 cm. Six-dimensional thermo-mechanical radar analyses revealed that S1 and S2 composites offer a balanced performance profile, combining low specific density, low thermal conductivity, and sufficient mechanical strength. In contrast, the CC1–CC2 and CS1–CS2 series excel in mechanical performance, while O1–O2 samples are characterized by higher density and thermal conductivity values.

Overall, the bio-composite insulation materials developed for the Kahramanmaraş climate demonstrate low density, controlled porosity, competitive thermal insulation performance, enhanced fire resistance, and significant CO₂ emission savings. These attributes make the materials a sustainable and energy-efficient alternative for building insulation applications in regions with similar climatic conditions.

## Data Availability

Data will be made available from the corresponding author upon reasonable request.
